# Effect of maternal foraging habitat on offspring quality in the loggerhead sea turtle (*Caretta caretta*)

**DOI:** 10.1002/ece3.3938

**Published:** 2018-02-27

**Authors:** Hideo Hatase, Kazuyoshi Omuta, Koutarou Itou, Teruhisa Komatsu

**Affiliations:** ^1^ Atmosphere and Ocean Research Institute The University of Tokyo Kashiwa Chiba Japan; ^2^ Yakushima Sea Turtle Research Group Yakushima Kagoshima Japan; ^3^ Minamichita Beachland Aquarium Mihama Aichi Japan; ^4^ Present address: Department of Commerce Yokohama College of Commerce Tsurumi, Yokohama Kanagawa Japan

**Keywords:** intrapopulation variation, migration, polymorphism, reptile, resource allocation

## Abstract

Exploring a trade‐off between quantity and quality of offspring allows differences in the fitness between alternative life histories to be accurately evaluated. We addressed the mechanism that maintains alternative life histories (small oceanic planktivores vs. large neritic benthivores) observed in a loggerhead sea turtle (*Caretta caretta*) population, which has been suggested to be environmental, based on the lack of genetic structure and a large difference in reproductive output. We examined whether maternal foraging habitat affects offspring quality, by measuring the morphology, emergence success, and righting response of hatchlings following incubation in a common open sand area over the whole nesting season at Yakushima Island, Japan, and by recording early growth and survival of offspring that were reared in a common environment at a Japanese aquarium. Furthermore, we tested whether sea turtles adjust egg size in response to temporal shifts of the incubation environment. There were no significant differences in any hatchling traits between oceanic and neritic foragers (which were classified by stable isotope ratios), except for clutches laid during the warmest period of the nesting season. There were also no significant differences in the growth and survival of offspring originating from the two foragers. The size of eggs from both foragers significantly increased as the season progressed, even though the rookery had heavy rainfall, negating the need to counteract heat‐related reduction in hatchling morphology. In comparison, the sizes of adult body and clutches from both foragers did not vary significantly. The results further support our previous suggestions that the size‐related foraging dichotomy exhibited by adult sea turtles does not have a genetic basis, but derives from phenotypic plasticity. Adjustment in reproductive investment may be associated with: (1) predation avoidance, (2) founder effect, and/or (3) annual variation in egg size.

## 1 INTRODUCTION

Intrapopulation variation in habitat use and its resultant alternative life histories are common in mobile animals (Bolnick et al., 2003). If they are based on genetics, these could be sources of biodiversity through sympatric population subdivision or speciation (Via, 2001). In contrast, if they are environmentally induced, they may function as bet‐hedging against wipeouts of fitness, due to the use of a single habitat in case of catastrophes (Krug, 2009). Thus, revealing the mechanisms that produce and maintain differential habitat use and alternative life histories in a population could advance our understanding of how organisms have survived and adapted during their evolutionary histories, and the forces that shape and maintain biodiversity. The exploration of genetic and fitness differences between alternative life histories is inevitable to address this topic, because these attributes are evolutionarily coupled; if alternative life histories have a genetic basis, fitness should be balanced; while if they are environmentally induced, fitness should be unequal (Gross, 1996).

Although technological advances in tracking have led to an increasing number of reports on differential habitat use and alternative life histories in populations with wide‐ranging distributions, the mechanisms that produce and maintain these variations have yet to be fully understood (Ceriani et al., 2015; Vander Zanden et al., 2014). For example, alternative life histories observed in a sea turtle population were suggested to be environmentally maintained, based on the genetic similarity at mitochondrial DNA sequences and microsatellite loci (Watanabe et al., 2011), and a large difference in offspring number produced between two alternative phenotypes (Hatase, Omuta, & Tsukamoto, 2013). The latter study used offspring number as a proxy for fitness, assuming that offspring quality was equivalent. However, if fewer offspring produced by one phenotype innately survive better until reaching sexual maturity than offspring produced by the other phenotype, differences in the productivity of the alternative phenotypes might be offset, possibly leading to balanced fitness. The low resolution of genetic markers used in the former study might have failed to detect differentiation between the alternative phenotypes, as found for a polymorphic fish (Skúlason, Snorrason, Noakes, & Ferguson, 1996). Although a trade‐off between quantity and quality of offspring, especially a trade‐off between egg size and number, has long been a topic of interest among sea turtle research communities (Bjorndal & Carr, 1989; LeBlanc et al., 2014; Wallace et al., 2007), it has not yet been demonstrated at the intrapopulation level, in contrast with other animal species (Gillespie, Russell, & Lummaa, 2008; Gustafsson & Sutherland, 1988; Khokhlova, Pilosof, Fielden, Degen, & Krasnov, 2014), where resource limitation is assumed. Regardless of the assumption of resource limitation, maternal food conditions do affect the quality of offspring (Annett & Pierotti, 1999; Kyneb & Toft, 2006). Thus, our current knowledge that alternative life histories observed in a sea turtle population are environmentally maintained might need reconsideration.

Aforementioned alternative sea turtle life histories are typically seen within some loggerhead turtle (*Caretta caretta*) populations breeding in Japan and Cape Verde; small adults, as well as juveniles, tend to forage on nutrient‐poor planktonic prey such as gelatinous zooplankton in oceanic waters (depth >200 m), while large adults tend to forage on nutrient‐rich benthic prey such as mollusks and crustaceans in neritic waters (depth <200 m) (Eder et al., 2012; Hatase, Matsuzawa, Sakamoto, Baba, & Miyawaki, 2002; Hatase, Omuta, & Tsukamoto, 2007; Hatase, Takai, et al., 2002; Hawkes et al., 2006; Varo‐Cruz et al., 2013). This size‐related foraging dichotomy does not imply that adults change habitat with age, at least within Japanese nesting populations, because (1) female loggerheads grow little after reaching sexual maturity (Hatase, Matsuzawa, Sato, Bando, & Goto, 2004) and (2) foraging habitats reflected in stable isotope ratios and remigration intervals (the intervals between successive nesting years) do not vary with breeding experience (Hatase, Takai, et al., 2002; Hatase et al., 2013). Rather, there is evidence that at least some of these adults would consistently use either oceanic or neritic habitat throughout a long span of their adult stage (Hatase et al., 2013).

Our previous studies suggested similar offspring quality from the nests of loggerhead turtles that forage in either oceanic or neritic habitat, with no significant differences in the size and nutritional components of eggs laid early in the nesting season (Hatase, Omuta, & Komatsu, 2014) and in the size and emergence success of hatchlings produced early in the nesting season (Hatase, Omuta, & Komatsu, 2015). However, because the incubation environment such as temperature and moisture at temperate rookeries shifts seasonally, experiments should encompass the whole nesting season. The incubation environment greatly affects the phenotype of offspring (Deeming & Ferguson, 1991). Thus, in this study, we examined whether maternal foraging habitat affects hatchling quality, which was measured as the morphology, emergence success, and righting response of hatchlings following the incubation of clutches in a common open sand area under a wide range of ambient temperatures. We also explored the effect of maternal foraging habitat on early growth and survival of offspring by rearing them in a common environment at an aquarium. By incubating clutches under similar environmental conditions in beach hatcheries and by rearing offspring under the same environment in an aquarium, environmental effect on offspring quality was separated from maternal effect. In particular, we focused on whether the offspring produced by oceanic foragers, which are 2.4‐fold fewer in quantity than those produced by neritic foragers (Hatase et al., 2013), have some advantage on survivability.

Furthermore, we examined maternal adjustment in reproductive investment within/among seasons. Ambient temperature fluctuates during the nesting season at temperate rookeries for sea turtles (Matsuzawa, Sato, Sakamoto, & Bjorndal, 2002), and the size of hatchlings produced during the warmer period of the nesting season is predicted to be smaller (e.g., Booth, 2017). As the survival of hatchlings may depend on body size (e.g., Janzen, 1993), smaller hatchlings produced during the warmer period of the nesting season may be disadvantageous. To counteract the heat‐related reduction in hatchling morphology, sea turtles that nest on temperate rookeries might invest more resources in individual eggs during the warmer period of the nesting season, because larger hatchlings generally hatch out from larger eggs (Pinckney, 1990; Wallace et al., 2007). Thus, we tested this hypothesis by examining seasonal/annual variations in egg size, in relation to variations in the sizes of adult body and clutches. Based on obtained results, we discuss how alternative life histories are maintained in a sea turtle population, and also how sea turtles cope with environmental changes.

## 2 MATERIALS AND METHODS

### 2.1 Surveys of nesting females and the relocation of their clutches

This study was conducted at the adjacent beaches of Inakahama (1.0 km in length), Maehama (0.9 km), and Yotsusehama (0.2 km) at Nagata (30°24′N, 130°26′E), Yakushima Island, Kagoshima Prefecture, Japan. Sea turtles generally nest from late April to early August on the island (Yakushima Sea Turtle Research Group 2011). To encompass the whole nesting season, nightly patrols looking for nesting turtles were conducted from 15 to 24 May 2013 (Hatase et al., 2014), 22 May to 5 June 2014 (Hatase et al., 2015), 25 June to 4 July 2015, and 4 to 9 July 2016. Although the nesting season continues until early August, it is difficult to obtain enough samples due to the decrease in nesting females late in the nesting season. Thus, we did not conduct sampling after early July. Adult female loggerhead turtles were individually identified by placing external plastic tags (MultiFlex P, Caisley, Bocholt, Germany) on both rear flippers and an internal passive integrated transponder (PIT) tag (ID‐100A Microtransponder, Trovan, East Yorkshire, UK) into the left front flipper. Their straight carapace lengths and widths were measured with calipers (Mantax Blue, Haglöf, Långsele, Sweden). Because there are fewer small oceanic foraging loggerheads than large neritic foragers at our study site (the ratio is 1:4; Hatase et al., 2013), small females with a straight carapace length and width of <810 and <633 mm, and large females with a straight carapace length and width of ≥810 and ≥630 mm were selected. These criteria allowed us to obtain similar sample sizes of small oceanic and large neritic foragers, which were classified from stable isotope ratios.

In 2013, five eggs per clutch of each female were collected from 10 small and 10 large females to analyze stable isotope and nutritional components (Hatase et al., 2014). Clutch size (the number of eggs laid in a nest) was not examined in 2013. The eggs were frozen and transported to our institute. The eggs were thawed and then weighed using a digital scale (FY‐300, A&D, Tokyo, Japan; accuracy ±0.01 g). During 2014–2016, one to three pairs of clutches laid by small and large females each night were collected to ensure they were subject to the same incubation environment. In total, 16 and 15 clutches laid by small and large females were collected in 2014 (one pair was not obtained due to a lack of a large female clutch), while clutches laid by 10 small and 10 large females were collected in each year of 2015 and 2016.

The 71 clutches laid by unique females during 2014–2016 were recovered from the nests during or within 6 hr of oviposition. At this time, clutch size was examined. The clutches were placed into plastic bags and transported to beach hatcheries, which were open sand areas located in high elevations, on Maehama beach in 2014 and 2015 and on Inakahama beach in 2016. Although the hatchery areas are guarded from visitors by ropes, they are naturally intruded and dug by nesting turtles. Five eggs per clutch were weighed with a digital scale (KP‐103, Tanita, Tokyo, Japan; accuracy ±0.3 g) in the field. One egg per clutch was collected for stable isotope analysis and kept at –20°C until analytical preparation. The scale used to weigh eggs was calibrated with a 100‐g weight before the onset of surveys each year. Clutches were reburied within artificial nests (a cylinder of 600 mm depth and 200 mm diameter) that were aligned in two or four rows with adjacent nests 0.8 m apart. Clutch size and the number of reburied eggs were different, because (1) some eggs were damaged during relocation and (2) one egg per clutch was collected for stable isotope analysis (see Table S1 in Supporting Information). Relocated nests were marked with plastic lines and numbered tags. Clutches were relocated within 7 hr of oviposition. The occupied areas were enclosed by stranded wood to defend against any intrusion by nesting turtles.

### 2.2 Surveys of hatchlings that emerged from the relocated nests

Incubation duration was defined as the number of days from the date of oviposition to the date of first observed emergence of hatchlings from the nest. Hatchlings were captured by covering relocated nests with plastic meshes from 4 to 17 August 2014, from 20 to 29 August 2015, and from 20 to 28 August 2016, based on previous estimates of the relationship between oviposition date and incubation duration for Japanese loggerhead turtles (Matsuzawa et al., 2002; Yakushima Sea Turtle Research Group 2011). The plastic meshes that covered relocated nests were checked every 1–2 hr from 1830 to 2200 and again at 0600 in 2014 and 2015, while they were checked overnight to assess the righting response of hatchlings in 2016 (see below). The plastic meshes were removed during the day to prevent emerging hatchlings from desiccating. When emerging hatchlings were observed within the meshes, the morphology of 2–17 hatchlings per nest was examined (see Table S1). Straight carapace lengths and widths of hatchlings were measured using digital calipers (CD‐15PSX, Mitutoyo, Kanagawa, Japan; accuracy ±0.02 mm), and their body mass was measured using a digital scale (the same one used for eggs) and a plastic cup on flat ground. The scale used to weigh hatchlings was calibrated with a 100‐g weight before initiating surveys of emerging hatchlings each year. The hatchlings sampled from some nests included hatchlings that emerged over several nights, because few hatchlings emerged during the first night. The morphology did not differ significantly between hatchlings that emerged on the first night and those that emerged on subsequent nights within nests (Hatase et al., 2015).

The ability of a hatchling to self‐right was assessed soon after it emerged from the nest in 2016, following the method of Booth, Feeney, and Shibata (2013). Each hatchling was placed on its carapace on a flat area of sand on the beach. Then, the time required for the hatchling to turn over onto its plastron was recorded with a stopwatch. If an individual took more than 10 s for a righting event, a 30‐s rest period (on the plastron) was given until the next attempt. These trials continued until three successful righting events were made, or until six unsuccessful attempts were made. A score from 0 to 6 indicating righting response propensity was given based on the numbers of trials and successful rightings: 0 for no righting event in six trials, 1 for one righting in six trials, 2 for two rightings in six trials, 3 for three rightings in six trials, 4 for three rightings in five trials, 5 for three rightings in four trials, and 6 for three rightings in three trials. The mean time for a hatchling to self‐right was calculated based on successful rightings. Six to ten hatchlings per nest were used in this righting response experiment (See Table S1). The hatchlings sampled from some nests included hatchlings that emerged over several nights, due to few hatchlings emerging on the first night.

The morphology and righting response of hatchlings were examined in the field during nights/mornings of mild weather, or under the top of a truck at Maehama beach or the roof of our research station at Inakahama beach on rainy nights/mornings. On nights with bad weather, the righting response of each hatchling was assessed inside a bucket that contained beach sand. Hatchlings captured at night were released soon after surveys; however, those captured in the morning were retained until dusk to prevent predation from crows and cats.

Emergence success was defined as the ratio of the number of hatchlings that emerged from a nest to the number of reburied eggs. Emergence success was recorded at 1830–2200 or at 0600 when excavating nests from which hatchlings had emerged 3–8 days earlier. Data on incubation duration, emergence success, and hatchling morphology for several nests were not obtained in 2014 and 2015, due to the destruction of a relocated nest by a nesting turtle or the absence of emerging hatchlings (Hatase et al., 2015).

### 2.3 Acquisition of environmental data

Mean sand temperature during incubation at nest depth was estimated using the regression equation between incubation temperature and duration for Japanese loggerheads (Matsuzawa et al., 2002): T = 639.8/D + 17.6, where T is the mean sand temperature during incubation, and D is incubation duration. Rainfall near Maehama beach was telemetered every 10 min by the municipality of Kagoshima Prefecture. Rainfall data during the incubation period of the experimental nests (i.e., between the start date of clutch relocation and the end date of first observed emergence of hatchlings from relocated nests) were summed for each year, and daily means were calculated. For comparison with Yakushima Island, rainfall data during the incubation season at other nesting sites, Ascension Island, UK, Black Rock, Trinidad, East Java, Indonesia, and Heron Island and Mon Repos, Australia, where incubation temperature affects hatchling morphology (Booth et al., 2013; Glen, Broderick, Godley, & Hays, 2003; Maulany, Booth, & Baxter, 2012; Mickelson & Downie, 2010; Sim, Booth, & Limpus, 2015), were obtained from relevant websites (See Table S2).

### 2.4 Rearing experiment

In 2016, 20 hatchlings (one hatchling per female) that were used to examine the morphology and righting response were kept for a rearing experiment. Nineteen hatchlings were collected on the first night of emergence from the nests, and one hatchling was collected on the second night of emergence. Righting response of one hatchling was not examined, although its morphology was examined. Hatchlings were individually identified by painting numbers on the carapaces. Collected hatchlings were kept in a polystyrene box with wet beach sand at our research station near Inakahama beach for one to 3 days until transportation. Three to ten hatchlings were placed in corrugated cartons lined with wet algae for transportation to an aquarium. It took one or 2 days for the packets of hatchlings to arrive at the aquarium. They were not fed during retention and transportation.

Twenty hatchlings were raised indoors (13‐hr light: 11‐hr dark) at the Minamichita Beachland Aquarium (34°47′N, 136°51′E), Mihama, Aichi Prefecture, Japan, from 26 August to 19 December 2016. Hatchlings were housed together within a 1700 × 660 mm mesh compartment with a water depth of 210 mm in a flow‐through seawater tank until 14 October. Thereafter, to prevent the offspring from biting each other, each offspring was housed singly within a 300‐mm‐diameter mesh compartment with a water depth of 210 mm in a flow‐through seawater tank. Mean (± *SD*) water temperature during the rearing period was 27.2 ± 1.0°C. They were fed pellets (made for rearing flatfish and pufferfish) four times daily until 7 October. Thereafter, they were fed a mixture of pellets (made for rearing soft‐shelled turtles) and minced mackerel and squid meat twice daily.

Growth of offspring was examined every 2 months, that is, on 31 October and 19 December. Straight carapace lengths and widths were measured using digital calipers (the same one used for hatchlings), and body mass was weighed using a digital scale (HL‐2000i; A&D, Tokyo, Japan; accuracy ±2 g). Unhealthy or dead turtles were removed from the rearing environment, and only data from live turtles were used for analysis of growth.

### 2.5 Measurements of stable isotope ratios and classification into foraging habitat groups

Stable carbon and nitrogen isotope ratios (δ^13^C and δ^15^N) in egg yolks were measured following Hatase, Takai, et al. (2002), Hatase, Sato, Yamaguchi, Takahashi, and Tsukamoto (2006). δ^13^C and δ^15^N were expressed as deviations from the standard, as defined by the following equation: δ^13^C or δ^15^N = (*R*_sample_/*R*_standard_ − 1) × 1000 (‰), where R is ^13^C/^12^C or ^15^N/^14^N. Vienna Pee Dee Belemnite (VPDB) and atmospheric nitrogen were used as the carbon and nitrogen isotope standards. Analytical precision was ≤0.19‰ for δ^13^C and ≤0.29‰ for δ^15^N. We classified females producing yolks with a δ^13^C of <–18.0‰ and a δ^15^N of <12.0‰ as oceanic planktivores and females with a δ^13^C of ≥−18.0‰ or a δ^15^N of ≥12.0‰ as neritic benthivores (Hatase et al., 2013, 2015; Watanabe et al., 2011) following the findings of a previous study that simultaneously conducted stable isotope analysis and satellite telemetry on the same females (Hatase, Takai, et al., 2002). Although it is difficult to classify turtles with isotopic values on and around the borders of these ranges accurately into the two foraging groups, we assumed that data of misclassified turtles were offset in the two groups during averaging. The complete data set for 2015 and 2016 is available as Supporting Information, including δ^13^C and δ^15^N in the yolks, body size, and the characteristics of eggs, hatchlings, and offspring derived from individual female loggerheads (See Tables [Link], [Link], [Link]).

### 2.6 Data analysis

Intraclutch means for the characteristics of eggs and hatchlings were used for analysis. Although the methods to measure egg mass were different between 2013 and later 3 years, data were merged. Parametric tests such as *t* tests and ANOVAs were used to compare the characteristics of adults, eggs, hatchlings, and offspring between oceanic and neritic foragers and among years. Welch's correction was added to unpaired *t* tests when variances were unequal. Emergence success (%) was arcsine‐transformed (°) before statistical tests.

Because several factors were considered to affect egg size and hatchling phenotype based on the results of the described tests, stepwise multiple regression analyses were conducted. Dependent variables were egg mass, in addition to the morphology, emergence success, and righting response of hatchlings. Foraging habitat, year, and adult straight carapace length were used as explanatory variables for egg mass during 2013–2016, with clutch size being an additional explanatory variable for egg mass during 2014–2016. Sand temperature, egg mass, and beach were used as alternative variables for hatchling morphology, in place of year and adult straight carapace length. Six variables were used for emergence success, including foraging habitat, egg mass, hatchling straight carapace length, clutch size, sand temperature, and beach. However, for righting response, beach was excluded as a variable from the six variables. Foraging habitat and beach were converted to dummy variables (oceanic = 0 and neritic = 1; Maehama = 0 and Inakahama = 1). Daily mean rainfall was not used as an explanatory variable in the analyses, because there was an illusory correlation between beach and daily mean rainfall due to small sample size. Explanatory variables with an *F*‐value of ≥4.0 were adopted as significant variables with forward selection.

## 3 RESULTS

### 3.1 Comparisons of adult and egg characteristics between foraging groups and among years

**Figure 1:**
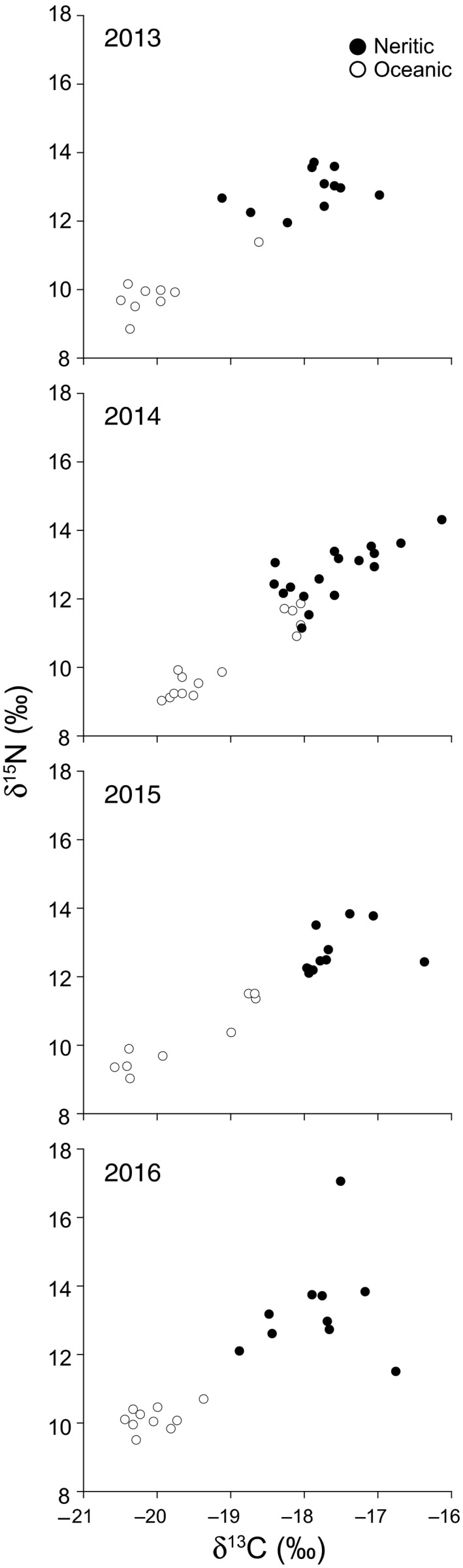
Plots of the δ^13^C and δ^15^N values in the yolks from eggs that were laid by 20, 31, 20, and 20 loggerhead turtles (*Caretta caretta*) at Yakushima Island, Japan, in 2013 (Hatase et al., 2014), 2014 (Hatase et al., 2015), 2015 (this study), and 2016 (this study), respectively. Turtles with a δ^13^C of <–18.0‰ and a δ^15^N of <12.0‰ were regarded as oceanic planktivores (open circles), while turtles with a δ^13^C of ≥−18.0‰ or a δ^15^N of ≥12.0‰ were regarded as neritic benthivores (filled circles)

Female loggerheads were divided into two groups based on the δ^13^C and δ^15^N values in egg yolks (Figure 1). There were nine oceanic planktivores and 11 neritic benthivores in 2013, 14 oceanic and 17 neritic foragers in 2014, nine oceanic and 11 neritic foragers in 2015, and 10 oceanic and 10 neritic foragers in 2016. The numbers of oceanic and neritic foragers in each year were similar to the numbers of small and large females that were sampled.

The straight carapace length and width of oceanic and neritic foragers differed significantly in all years (Table 1). Oceanic foragers had shorter mean straight carapace lengths and widths than neritic ones. The straight carapace lengths and widths of adults did not vary with year (Table 1). Clutch size varied significantly between the two foraging groups in all 3 years, with oceanic foragers laying smaller clutches than neritic ones (Table 1). Similarly, the mean number of eggs that were reburied per nest for oceanic foragers was significantly smaller than that for neritic ones in all 3 years (See Table S1). Although clutch size did not vary significantly with year, it became smaller in later years (Table 1).

**Table 1:** Comparisons of body size and egg and hatchling characteristics between oceanic and neritic foraging loggerhead turtles (*Caretta caretta*) nesting at Yakushima Island, Japan, during 2013–2016 (Hatase et al., 2014, 2015; this study). Nesting females were surveyed at different periods of the nesting season: 15 to 24 May 2013, 22 May to 5 June 2014, 25 June to 4 July 2015, and 4 to 9 July 2016. Clutches were relocated at Maehama beach in 2014 and 2015 and at Inakahama beach in 2016. Turtles were separated into the two foraging groups based on δ^13^C and δ^15^N in egg yolks. *n* is sample size. Significant *p* values are presented in bold. See Table S1 for additional data

Parameter, by year	Oceanic			Neritic			Unpaired *t* test
	Mean ± *SD*	Range	*n*	Mean ± *SD*	Range	*n*	*p*
Adult female							
Straight carapace length (mm)							
2013	789 ± 47	757–908	9	852 ± 65	759–918	11	**.027**
2014	783 ± 38	727–897	14	860 ± 51	754–942	17	**<.0001**
2015	788 ± 38	750–877	9	866 ± 41	795–923	11	**<.0005**
2016	759 ± 22	725–790	10	848 ± 15	827–874	10	**<.0001**
two‐way ANOVA: ***p *** **<** *** *** **.0001** for forager, *p *=* *.361 for year, *p *=* *.811 for interaction							
Straight carapace width (mm)							
2013	622 ± 33	584–683	9	667 ± 39	610–719	11	**.013**
2014	615 ± 25	590–691	14	676 ± 36	607–732	17	**<.0001**
2015	618 ± 21	590–668	9	676 ± 40	587–720	11	**<.001**
2016	605 ± 20	558–628	10	667 ± 21	630–690	10	**<.0001**
two‐way ANOVA: ***p *** **<** *** *** **.0001** for forager, *p *=* *.679 for year, *p *=* *.786 for interaction							
Egg							
Clutch size (number of eggs laid per nest)							
2014	100.4 ± 12.7	83–121	14	123.6 ± 16.4	93–149	17	**<.0005**
2015	99.6 ± 11.0	86–122	9	124.6 ± 25.2	77–162	11	**.010**
2016	94.0 ± 13.9	77–119	10	118.4 ± 14.8	97–144	10	**<.005**
two‐way ANOVA: ***p *** **<** *** *** **.0001** for forager, *p *=* *.405 for year, *p *=* *.978 for interaction							
Mass (g)^a^							
2013	27.5 ± 2.9	22.8–32.8	9	29.7 ± 3.8	24.9–36.4	11	.170
2014	30.7 ± 2.1	27.3–34.7	14	31.3 ± 4.4	22.9–37.4	17	.623
2015	33.9 ± 3.8	29.3–41.2	9	34.1 ± 2.3	30.5–38.0	11	.891
2016	30.8 ± 3.8	25.7–38.1	10	34.1 ± 2.1	31.0–37.4	10	**.031**
two‐way ANOVA: ***p *** **=** *** *** **.031** for forager, ***p *** **<** *** *** **.0001** for year, *p *=* *.403 for interaction							
Hatchling							
Incubation duration (days)							
2014	69.8 ± 1.9	66–74	11	69.9 ± 2.6	66–75	17	.895
2015	55.8 ± 2.0	52–58	8	56.0 ± 2.5	52–61	10	.823
2016	48.2 ± 0.9	47–49	10	48.1 ± 1.2	46–50	10	.836
Estimated mean sand temperature during incubation at nest depth (°C)							
2014	26.8 ± 0.3	26.3–27.3	11	26.8 ± 0.3	26.1–27.3	17	
2015	29.1 ± 0.4	28.6–29.9	8	29.0 ± 0.5	28.1–29.9	10	
2016	30.9 ± 0.3	30.7–31.2	10	30.9 ± 0.3	30.4–31.5	10	
Straight carapace length (mm)^b^							
2014	40.46 ± 1.44	38.14–42.50	12	40.39 ± 1.69	37.29–43.74	17	.905
2015	41.32 ± 1.27	39.43–42.85	8	41.50 ± 1.00	40.04–42.96	10	.734
2016	40.33 ± 1.40	38.12–42.45	10	41.90 ± 0.88	40.57–43.20	10	**.007**
two‐way ANOVA: *p *=* *.106 for forager, ***p *** **=** *** *** **.046** for year, *p *=* *.110 for interaction							
Straight carapace width (mm)^b^							
2014	32.43 ± 1.15	31.10–34.98	12	32.84 ± 1.36	29.96–34.64	17	.401
2015	33.43 ± 0.93	31.92–34.92	8	33.49 ± 0.74	32.12–34.86	10	.887
2016	32.60 ± 1.14	30.35–34.06	10	33.80 ± 0.63	32.61–34.60	10	**.009**
two‐way ANOVA: ***p *** **=** *** *** **.044** for forager, ***p *** **=** *** *** **.035** for year, *p *=* *.245 for interaction							
Body mass (g)^b^							
2014	15.3 ± 1.6	13.3–18.7	12	15.6 ± 2.1	11.7–19.1	17	.739
2015	16.4 ± 1.2	15.1–17.9	8	16.9 ± 1.0	15.1–18.2	10	.309
2016	15.0 ± 1.9	12.3–17.9	10	17.1 ± 1.1	15.7–19.1	10	**.008**
two‐way ANOVA: ***p*** ** = .023** for forager, *p *=* *.058 for year, *p *=* *.144 for interaction							
Emergence success (%)^c^							
2014	40.7 ± 25.1	0–85.1	13	44.6 ± 15.0	19.5–68.9	17	.499
2015	37.0 ± 20.7	0–68.8	9	31.0 ± 26.4	0–80.3	11	.629
2016	68.8 ± 10.4	51.7–83.9	10	55.6 ± 12.0	41.3–79.2	10	**.018**
Righting response propensity^d^							
2016	5.4 ± 0.8	3.2–6.0	10	5.0 ± 0.5	4.2–5.6	10	.150
Righting response time (s)^d^							
2016	2.51 ± 0.32	2.10–3.07	10	2.71 ± 0.52	2.02–3.75	10	.303

a Mean values among five eggs per clutch were used for calculations.

b Mean values among 2–17 hatchlings per nest were used for calculations.

c Arcsine‐transformed values were used for statistical tests.

d Mean values among 6–10 hatchlings per nest were used for calculations.

There were no significant differences in egg mass between the two foraging groups in 2013, 2014, or 2015, while eggs laid by oceanic foragers in 2016 were significantly lighter than those laid by neritic ones (Table 1). Egg mass significantly differed among years (Table 1), with mean egg masses in 2013 and 2014 being lighter than those in other years (Fisher's PLSD: *p *=* *.019 for 2013 vs. 2014, *p *<* *.0001 for 2013 vs. 2015, *p *<* *.001 for 2013 vs. 2016, and *p *=* *.002 for 2014 vs. 2015).

### 3.2 Comparisons of hatchling characteristics between foraging groups and among years

Incubation durations were not significantly different between oceanic and neritic foragers in all 3 years (Table 1). Incubation durations shortened with year due to an increase in sand temperature (Table 1). Like the differences in egg mass between oceanic and neritic foragers, no significant differences were found in straight carapace length, straight carapace width, and body mass of hatchlings between the two foragers in 2014 and 2015, while those of hatchlings from oceanic foragers were significantly shorter and lighter than those from neritic ones in 2016 (Table 1). Like annual variation in egg mass, there were significant differences in hatchling straight carapace length and width among years (Table 1), with mean straight carapace length and width in 2014 being shorter than those in 2015 (Fisher's PLSD: *p *=* *.017 for length, *p *=* *.017 for width). Although hatchling body mass also varied among years, these differences were not significant (Table 1).

Emergence success was not significantly different between the two foraging groups in 2014 and 2015, while that from oceanic foragers in 2016 was significantly higher than that from neritic ones (Table 1). Emergences success of both oceanic and neritic foragers in 2016 was higher than that of previous 2 years (Table 1), probably because incubation environment such as sand characteristics on a hatchery at Inakahama beach was better than that at Maehama beach (Yakushima Sea Turtle Research Group 2011), which was reflected in a result of multiple regression analysis (see the next section). No significant differences were found for righting response propensity or time between hatchlings that emerged on the first night and those that emerged on subsequent nights within six nests (paired *t* tests: *p *=* *.722 or .672). There were no significant differences in righting response propensity or time of hatchlings between the two foragers (Table 1).

For experimental nests, rainfall during the incubation period was 1584 mm over 84 days (i.e., 18.9 mm/day) in 2014, 1509 mm over 66 days (22.9 mm/day) in 2015, and 474 mm over 55 days (8.6 mm/day) in 2016. Daily mean rainfall during these 3 years did not vary predictably like the seasonal increase in sand temperature (Table 1). Daily mean rainfall during the incubation season at other nesting sites was much lower: 0.4 mm/day at Ascension Island, UK, 3.8 mm/day at Black Rock, Trinidad, 2.2 mm/day at East Java, Indonesia, 3.7 mm/day at Heron Island, Australia, and 5.1 mm/day at Mon Repos, Australia (See Table S2).

### 3.3 Multiple regression analyses

Year (positive) and adult straight carapace length (positive) significantly affected egg mass during 2013–2016, with clutch size (negative) also being adopted as a significant explanatory variable for egg mass during 2014–2016 (Table 2). Only egg mass (positive) significantly affected straight carapace length, straight carapace width, and body mass of hatchlings (Table 2). Clutch size (negative) and beach significantly affected emergence success (Table 2). Although clutch size (negative) was adopted as a significant explanatory variable for righting response propensity, *R* ^2^ was not significantly different from 0 (Table 2). None of five variables significantly affected righting response time (Table 2). Foraging habitat did not affect any dependent variables (Table 2).

**Table 2:** Stepwise multiple regression analyses of egg and hatchling characteristics derived from loggerhead turtles (*Caretta caretta*) nesting at Yakushima Island, Japan, during 2013–2016. Adopted explanatory variables have regression coefficients, standardized regression coefficients, and *F*‐values (≥4.0)

Dependent variable	Explanatory variable	Regression coefficient	Standardized regression coefficient	*F*‐value	*R* ^2^ and *p*‐value based on adopted explanatory variables
Egg mass during 2013–2016^a^	Year	1.567	0.440	26.0	.352 (*p *<* *.0001)
	Adult straight carapace length	0.029	0.446	26.7	
	F				
Egg mass during 2014–2016	Year	1.071	0.255	6.3	0.329 (*p *<* *.0001)
	Adult straight carapace length	0.048	0.764	27.7	
	Clutch size	–0.078	–0.444	9.4	
	F				
Hatchling straight carapace length	Egg mass	0.339	0.793	109.8	.628 (*p *<* *.0001)
	F, C, T, and B				
Hatchling straight carapace width	Egg mass	0.264	0.783	102.9	.613 (*p *<* *.0001)
	F, C, T, and B				
Hatchling body mass	Egg mass	0.445	0.866	195.5	.750 (*p *<* *.0001)
	F, C, T, and B				
Emergences success^b^	Clutch size	–0.165	–0.270	6.1	.277 (*p *<* *.0001)
	Beach	10.728	0.402	13.5	
	F, E, T, and H				
Righting response propensity	Clutch size	–0.016	–0.427	4.0	.183 (*p *=* *.060)
	F, E, T, and H				
Righting response time	F, E, C, T, and H				0 (undetermined *p*)

Abbreviations are explanatory variables that were not adopted: F for foraging habitat, E for egg mass, C for clutch size, T for sand temperature, B for beach, and H for hatchling straight carapace length

a Clutch size was not examined in 2013.

b Arcsine‐transformed values were used for analysis.

### 3.4 Comparisons of growth and survival of offspring between foraging groups

Of the 20 hatchlings kept for a rearing experiment, an equal number (10) were from oceanic and neritic foraging females. Although there were no significant differences in straight carapace length (unpaired *t* test: *p *=* *.112) or width (*p *=* *.598) in hatchlings produced by oceanic and neritic foragers, their body mass differed significantly (*p *=* *.024). The mean body mass of hatchlings produced by oceanic foragers was lighter than that of hatchlings from neritic foragers, possibly a result of the lighter eggs laid by oceanic foragers in 2016 (Table 1). There were no significant differences in righting response propensity (unpaired *t* test: *p *=* *.557) or time (unpaired *t* test with Welch's correction: *p *=* *.113) between hatchlings produced by oceanic and neritic foragers.

**Figure 2:**
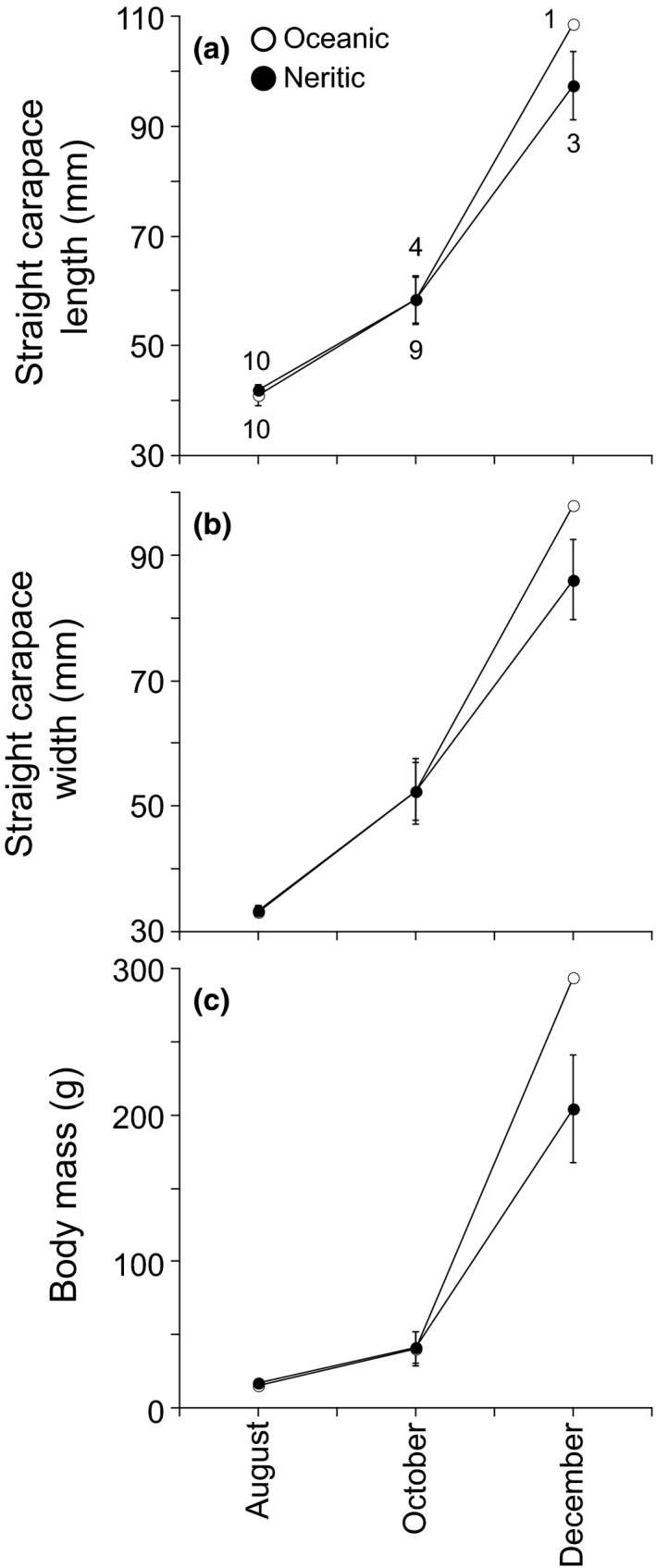
Growth of (a) straight carapace length, (b) straight carapace width, and (c) body mass for offspring produced by either oceanic (open circle) or neritic (filled circle) foraging loggerhead turtles (*Caretta caretta*), reared in a common environment. Maternal foraging areas were distinguished based on δ^13^C and δ^15^N in yolks of eggs from the source clutches. Symbols and error bars are means ± *SD*. Numbers above and below symbols are sample sizes of live offspring produced by oceanic and neritic foragers. Sample sizes are identical in panels (a–c)

Of the 20 hatchlings raised, four and nine from oceanic and neritic foragers survived until 31 October 2016. There were no significant differences in the frequencies of live and dead offspring between the two foragers (Fisher's exact test: *p *=* *.057). Live offspring derived from both foragers grew similarly with respect to straight carapace lengths, straight carapace width, and body mass (two‐way repeated‐measures ANOVA: *p *=* *.807 for length, *p *=* *.904 for width, *p *=* *.937 for mass; Figure 2).

One and three offspring from oceanic and neritic foragers survived until 19 December 2016. Although statistical tests could not be performed on growth data due to the small sample size, the offspring grew similarly (Figure 2). There were no significant differences in the frequencies of live and dead offspring between the two foragers (Fisher's exact test: *p *=* *.582).

## 4 DISCUSSION

The current study showed that differences in the use of foraging habitats by adult female sea turtles do not affect the quality of hatchlings incubated in a common open sand area and of offspring reared under the same environment, except for the quality of hatchlings produced during the warmest period of the nesting season. At the warmest period, hatchlings derived from oceanic foragers were significantly smaller and lighter, with significantly higher emergence success. However, righting response of hatchlings was similar in both foragers. These phenotypic differences may be attributed to differences in maternal investment on eggs and clutches, rather than to the differential development process of embryos and hatchlings under a common incubation environment (see the next paragraph). Because the survival of hatchlings may depend on body size (Janzen, 1993), smaller and lighter hatchlings produced by oceanic foragers during the warmest period may be disadvantageous. In addition, the higher emergence success of hatchlings from oceanic foragers is offset by smaller clutch size, resulting in a similar number of hatchlings emerging per nest during the warmest period for the two foragers. Thus, the survival rate during the period from aboveground emergence to first reproduction for offspring derived from oceanic foragers would not be high enough to offset their producing 2.4‐fold fewer offspring than neritic ones. Fitness would thus not be balanced between the two foragers. These findings support our previous suggestions that the size‐related foraging dichotomy exhibited by adult sea turtles does not have a genetic basis, but is derived from phenotypic plasticity (Hatase et al., 2013; Watanabe et al., 2011). A trade‐off between quantity and quality of offspring does not seem to occur in a large marine reptile, in contrast with the cases of other animal species (Gillespie et al., 2008; Gustafsson & Sutherland, 1988; Khokhlova et al., 2014), where resource limitation was assumed.

Maternal body size sometimes determines the quantity of resources allocated to the size and number of offspring, ultimately influencing the quality of offspring. Eggs and hatchlings produced during the warmest period of the nesting season (i.e., in 2016) were significantly different between small oceanic and large neritic foraging loggerheads. Oceanic foragers produced significantly smaller eggs than neritic ones in 2016, possibly due to smaller body size of sampled oceanic foragers in that year than that of previous 3 years. This is because larger female loggerheads lay larger eggs at Yakushima Island (Hatase et al., 2015) in contrast with other nesting sites (Tiwari & Bjorndal, 2000). In addition, because larger hatchlings hatch out from larger eggs (Hatase et al., 2015), hatchlings derived from oceanic foragers were significantly smaller and lighter than those derived from neritic ones in 2016. A multiple regression analysis indeed implied that hatchling morphology is determined only by egg mass at Yakushima Island. The significantly lower emergence success of hatchlings derived from neritic foragers compared to oceanic ones in 2016 may be that larger clutches derived from larger neritic foragers were more negatively impacted by metabolic heat and hypoxia within nests during the warmest period. This is because clutch size is correlated positively with temperature and negatively with oxygen content within sea turtle nests (Chen, Wang, & Cheng, 2010; Wallace et al., 2004). This inference was consistent with the negative effect of clutch size on emergence success in a multiple regression analysis. In the analysis, sand temperature was not adopted as an explanatory variable for emergence success, possibly because sand temperature and nest temperature act differently.

Maternal and environmental factors synergistically shape offspring phenotype. Irrespective of foraging habitat, female loggerheads laid larger eggs during the warmer period of the nesting season at Yakushima Island. In general, smaller sea turtle hatchlings emerge from clutches incubated under warmer temperatures (e.g., Booth, 2017). Thus, similar‐sized loggerhead hatchlings were expected to emerge throughout the nesting season, due to larger egg size being offset by warmer temperature. However, parallel to the seasonal increase in egg size, larger hatchlings emerged during the warmer period of the nesting season. This result suggests that incubation temperature does not affect the morphology of loggerhead hatchlings at Yakushima Island. In fact, a multiple regression analysis supported this inference. As moisture also affects hatchling morphology in turtles (Hewavisenthi & Parmenter, 2001; Packard, 1999), the absence of a thermal effect on hatchling morphology in our study may be due to heavy rainfall on the island, which kept sand moist around nests, possibly buffering heat‐related reduction in hatchling morphology. In fact, rainfall during the incubation season at nesting sites where hatchling morphology was affected by incubation temperature (Ascension Island, UK: Glen et al., 2003; Black Rock, Trinidad: Mickelson & Downie, 2010; East Java, Indonesia: Maulany et al., 2012; Heron Island, Australia: Booth et al., 2013; Mon Repos, Australia: Sim et al., 2015) was much lower (0.4–5.1 mm/day) than that at Yakushima Island (8.6–22.9 mm/day).

Why do loggerheads lay larger eggs during the warmer period of the nesting season on Yakushima Island where a thermal effect on hatchling morphology is negligible? This may be that eggs sampled in later years (i.e., during the warmer period of the nesting season) were from larger females, because egg size is proportional to female body size at Yakushima Island (Hatase et al., 2015). Although the lack of significant differences in female body size among years did not support this inference, a multiple regression analysis showed that both year (i.e., nesting period) and female body size were responsible for egg mass. Thus, the annual/seasonal increase in egg mass was partly attributed to an annual/seasonal increase in female body size. The absence of significant differences in clutch size among years suggests that Yakushima loggerheads lay larger eggs later in the nesting season without expensing clutch size, although clutch size slightly decreased in later years (i.e., later in the nesting season). This trend was consistent with a multiple regression analysis. The seasonal decrease in clutch size has often been attributed to resource depletion occurring later in the nesting season among other loggerhead turtle populations (Broderick, Glen, Godley, & Hays, 2003; Frazer & Richardson, 1985; LeBlanc et al., 2014). However, a trade‐off between clutch size and egg size might underlie this trend.

We propose three hypotheses for the seasonal increase in egg size among loggerheads nesting at Yakushima Island: (1) predation avoidance, (2) founder effect, and/or (3) annual variation in egg size. The need to avoid predators may increase as the incubation season progresses. Because larger hatchlings may escape from predators better (Janzen, 1993), Yakushima loggerheads may produce larger eggs and hatchlings later in the nesting season. To test this hypothesis, surveys on the seasonal occurrence of terrestrial and marine predators are needed. Alternatively, this egg size tendency may have been inherited from a founder that first colonized Yakushima Island. Japanese loggerheads, which nest on temperate beaches with seasonally fluctuating sand temperature, may have evolved such a trait to compensate for heat‐related reduction in hatchling morphology during the warmer period of the nesting season. Whether similar seasonal shifts in egg size are observed on other Japanese loggerhead nesting sites should be verified to confirm this hypothesis. Finally, the observed “seasonal” increase in egg size might simply reflect annual variation in egg size. However, because eggs laid by females using different foraging habitats are not likely to exhibit similar annual variations in size, this trend may truly be seasonal. Future studies should weigh eggs throughout one nesting season to confirm within‐season variation in egg size. There are few studies that report seasonal and annual variations in egg size among sea turtles; egg size of green turtles (*Chelonia mydas*) did not vary seasonally at Tortuguero, Costa Rica (Bjorndal & Carr, 1989), whereas egg size of loggerhead turtles on Cephalonia, Greece (Hays & Speakman, 1992), and Georgia, the United States (LeBlanc et al., 2014) reduced seasonally due to possible resource depletion. Although loggerhead egg size in Georgia, the United States, showed no annual variation (LeBlanc et al., 2014), egg size of some freshwater turtles did vary annually (Rowe, 1994; Schwarzkopf & Brooks, 1986). Thus, sea turtles might also be able to adjust egg size more drastically in response to the local environment than previously thought, as shown in the present study.

Common garden experiments are frequently conducted to verify whether a polymorphism is shaped by intrinsic factors such as genetics (e.g., Ortega, López, & Martín, 2015). For example, progeny of four sympatric morphs of the Icelandic Arctic char (*Salvelinus alpinus*) were reared in a common environment but grew differently and matured at different ages, confirming a genetic basis to their life history variations (Skúlason et al., 1996). In contrast, it is difficult to raise long‐lived sea turtles to sexual maturity in a common environment. Thus, early growth trajectories and survivorship may be more practical indices for verifying whether life history variations are genetically driven within a sea turtle population. Although we did not detect significant differences in growth rate and survival rate of offspring between oceanic and neritic foraging loggerheads, the sample size and rearing period were not sufficient to confidently deny a genetic basis to the foraging dichotomy. Thus, the results of the rearing experiment should be viewed with caution. Future studies should increase sample size and improve the techniques to raise offspring.

In conclusion, the present study further supported the environmental maintenance of alternative life histories in a sea turtle population. However, to evaluate the fitness differences between alternative life histories accurately, additional early life history traits must be investigated. Although the present study focused mainly on the morphology and emergence success of hatchlings, their terrestrial and aquatic locomotor performance should also be assessed (Sim et al., 2015). Crawling and swimming performance may reflect hatchling quality more accurately than righting response (Fisher, Godfrey, & Owens, 2014). In addition, sex differences in offspring characteristics should be taken into account. Sexes of sea turtles are determined by incubation temperature; temperatures higher than about 29°C produce females, while lower temperatures produce males (Ackerman, 1997). Because estimated mean incubation temperatures for the hatchlings used for righting response and rearing experiments were 30.4–31.5°C, all hatchlings were presumably females. Future studies should include male hatchlings, which are produced early in the nesting season. Furthermore, microhabitat selection by females on nesting beaches and its fitness consequences (Kamel & Mrosovsky, 2004) should be compared between the two foragers.

## CONFLICT OF INTEREST

None declared.

## AUTHOR CONTRIBUTIONS

HH and TK conceived and designed this study. HH and KO conducted field surveys. KI raised turtles. HH performed stable isotope analysis and analyzed the data to write the manuscript. All authors contributed critically to the drafts and gave final approval for publication.

## DATA ACCESSIBILITY

The original data on adult, egg, and hatchling characteristics in 2013 and 2014 are available in electronic supplementary materials for other journals (https://doi.org/10.1016/j.jembe.2014.03.002 and https://doi.org/10.1007/s00227-015-2693-x). The original data on the characteristics of adults, eggs, hatchlings, and offspring in 2015 and 2016 are uploaded as online supporting information.
