# Supplementary material for: Effect of maternal foraging habitat on offspring quality in the loggerhead sea turtle (Caretta caretta)
Source: Ecol Evol. 2018 Feb 27;8(6):3543–55. doi: 10.1002/ece3.3938 (PMC5869213; doi:10.1002/ece3.3938)
Supplement: Supplementary file 1 [file ECE3-8-3543-s001.pdf]

Table S1. Additional data on the numbers of eggs and hatchlings derived from oceanic and neritic foraging loggerhead turtles (*Caretta caretta)* nesting at Yakushima Island, Japan, during 2014–2016. Turtles were separated into the two foraging groups based on $\delta^{13}C$ and $\delta^{15}N$ in egg yolks. *n* is sample size.

Parameter, by year	Oceanic			Neritic			unpaired *t*-test
	Mean ± SD	Range	*n*	Mean ± SD	Range	*n*	*P*
**Egg**							
Number of eggs reburied per nest							
2014	98.9 ± 13.3	79–120	14	121.2 ± 16.3	91–148	17	<0.0005
2015	97.8 ± 11.0	83–119	9	121.8 ± 24.0	76–157	11	0.010
2016	93.0 ± 13.9	76–118	10	117.2 ± 14.8	96–143	10	<0.005
**Hatchling**							
Number of hatchlings sampled per nest for measuring morphology							
2014	8.2 ± 2.8	2–11	12	9.1 ± 1.6	6–11	17	
2015	10	10	8	9.4 ± 1.3	6–10	10	
2016	10.6 ± 1.9	10–16	10	10.7 ± 2.2	10–17	10	
Number of hatchlings sampled per nest for assessing righting response							
2016	9.6 ± 1.3	6–10	10	9.7 ± 0.9	7–10	10	

Effect of maternal foraging habitat on offspring quality in the loggerhead sea turtle (*Caretta caretta*)
Ecology and Evolution
Hideo Hatase*, Kazuyoshi Omuta, Koutarou Itou and Teruhisa Komatsu
*Corresponding author: hhatase@yahoo.co.jp
