# Supplementary material for: Effect of maternal foraging habitat on offspring quality in the loggerhead sea turtle (Caretta caretta)
Source: Ecol Evol. 2018 Feb 27;8(6):3543–55. doi: 10.1002/ece3.3938 (PMC5869213; doi:10.1002/ece3.3938)
Supplement: Supplementary file 2 [file ECE3-8-3543-s002.pdf]

Table S2. Sources of rainfall data during the incubation season on other sea turtle nesting sites, where incubation temperature affected hatchling morphology

Site	Incubation season	Daily mean rainfall (mm)	URL	Accessed	Reference
Ascension Island, UK	December to August	0.4	http://www.ascension.climatemps.com/precipitation.php	26 December 2016	Godley et al. (2002); Glen et al. (2003)
Black Rock, Trinidad	April to Jun	3.8	https://en.climate-data.org/location/123874/	02 February 2017	Mickelson & Downie (2010)
East Java, Indonesia	April to August	2.2	http://www.meteovista.com/Asia/Indonesia/Banyuwangi/4703539 #ui-tabs-14	02 February 2017	Maulany et al. (2012)
Heron Island, Australia	December to February	3.7	http://www.bom.gov.au/climate/averages/tables/cw_039122.shtml	26 December 2016	Booth et al. (2013)
Mon Repos, Australia	December to February	5.1	http://www.bom.gov.au/climate/averages/tables/cw_039128.shtml	02 February 2017	Sim et al. (2015)

References:

Booth, D.T., Feeney, R. & Shibata, Y. (2013) Nest and maternal origin can influence morphology and locomotor performance of hatchling green turtles (*Chelonia mydas*) incubated in field nests. *Marine Biology* , **160**, 127–137.

Glen, F., Broderick, A.C., Godley, B.J. & Hays, G.C. (2003) Incubation environment affects phenotype of naturally incubated green turtle hatchlings. *Journal of the Marine Biological Association of the United Kingdom* , **83**, 1183–1186.

Godley, B.J., Broderick, A.C., Frauenstein, R., Glen, F. & Hays, G.C. (2002) Reproductive seasonality and sexual dimorphism in green turtles. *Marine Ecology Progress Series* , **226**:125–133.

Maulany, R.I., Booth, D.T. & Baxter, G.S. (2012) The effect of incubation temperature on hatchling quality in the olive ridley turtle, *Lepidochelys olivacea* , from Alas Purwo National Park, East Java, Indonesia: implications for hatchery management. *Marine Biology* , **159**, 2651–2661.

Mickelson, L.E. & Downie, J.R. (2010) Influence of incubation temperature on morphology and locomotion performance of Leatherback (*Dermochelys coriacea*) hatchlings. *Canadian Journal of Zoology* , **88**, 359–368.

Sim, E.L., Booth, D.T. & Limpus, C.J. (2015) Incubation temperature, morphology and performance in loggerhead (*Caretta caretta*) turtle hatchlings from Mon Repos, Queensland, Australia. *Biology Open* , **4**, 685–692.

Effect of maternal foraging habitat on offspring quality in the loggerhead sea turtle (*Caretta caretta*)
Ecology and Evolution
Hideo Hatase*, Kazuyoshi Omuta and Teruhisa Komatsu
*Corresponding author: hhatase@yahoo.co.jp
