# Supplementary material for: Effect of maternal foraging habitat on offspring quality in the loggerhead sea turtle (Caretta caretta)
Source: Ecol Evol. 2018 Feb 27;8(6):3543–55. doi: 10.1002/ece3.3938 (PMC5869213; doi:10.1002/ece3.3938)
Supplement: Supplementary file 3 [file ECE3-8-3543-s003.pdf]

Table S3. The original data on stable carbon and nitrogen isotope ratios ($\delta^{13}C$ and $\delta^{15}N$) in egg yolks, body size, and egg and hatchling characteristics for 20 loggerhead turtles (*Caretta care*[illegible]

Tag number	Tag number	Oviposition date	$\delta^{13}C$ (‰)	$\delta^{15}N$ (‰)	Foraging habitat	Adult straight carapace length (mm)	Adult straight carapace width (mm)	Cltuch size	Number of eggs reburied	Number of emerging hatchlings	Emergence success (%)	Mass (g) for 5 eggs		Date of first emergence	Date of excavation survey	Incubation duration
												Mean	CV			
Y5670	Y5671	3-Jul	-20.37	9.04	Oceanic	768	620	96	95	34	35.79	29.28	0.0803	24-Aug	27-Aug	52
Y5403	Y5404	3-Jul	-20.58	9.37	Oceanic	790	615	97	96	66	68.75	34.76	0.0172	28-Aug	31-Aug	56
Y5560	Y5561	26-Jun	-20.41	9.40	Oceanic	750	590	88	87	13	14.94	32.06	0.0332	23-Aug	27-Aug	58
Y5164	Y5165	4-Jul	-19.93	9.69	Oceanic	795	615	94	90	51	56.67	36.92	0.0291	28-Aug	31-Aug	55
Y5551	Y5548	26-Jun	-20.38	9.91	Oceanic	753	612	86	83	0	0.00	41.22	0.015	–	28-Aug	–
Y5355	Y5365	29-Jun	-19.00	10.40	Oceanic	790	618	122	119	53	44.54	29.9	0.0316	23-Aug	27-Aug	55
Y5606		27-Jun	-18.66	11.38	Oceanic	800	605	103	102	46	45.10	35.2	0.0308	23-Aug	28-Aug	57
Y5047	Y5046	25-Jun	-18.76	11.51	Oceanic	766	617	102	101	39	38.61	31.34	0.0302	22-Aug	26-Aug	58
Y5590	Y5591	26-Jun	-18.68	11.53	Oceanic	877	668	108	107	31	28.97	34.62	0.0165	20-Aug	25-Aug	55
Y5326	Y5327	29-Jun	-17.95	12.11	Neritic	807	587	103	102	35	34.31	35.28	0.0226	23-Aug	27-Aug	55
Y5711	Y5713	29-Jun	-17.89	12.22	Neritic	914	698	144	141	29	20.57	34.88	0.0177	23-Aug	27-Aug	55
Y5640	Y5639	29-Jun	-17.95	12.25	Neritic	867	688	135	134	12	8.96	32.82	0.0153	29-Aug	1-Sep	61
Y5701	Y5702	3-Jul	-17.97	12.27	Neritic	857	691	122	120	7	5.83	30.46	0.024	26-Aug	31-Aug	54
Y5329		27-Jun	-16.38	12.45	Neritic	879	710	134	131	35	26.72	34.08	0.0328	23-Aug	27-Aug	57
Y4887	Y4889	26-Jun	-17.80	12.49	Neritic	836	658	114	111	0	0.00	37.6	0.0647	–	28-Aug	–
Y5615	Y5616	30-Jun	-17.71	12.51	Neritic	795	625	77	76	61	80.26	31.76	0.0433	27-Aug	30-Aug	58
Y5167	Y5533	30-Jun	-17.68	12.80	Neritic	923	700	107	105	17	16.19	38.02	0.068	24-Aug	28-Aug	55
Y4616		25-Jun	-17.85	13.52	Neritic	895	720	162	157	50	31.85	32.26	0.0392	22-Aug	26-Aug	58
99214	Y5693	4-Jul	-17.07	13.78	Neritic	892	692	158	152	62	40.79	34.28	0.0342	25-Aug	29-Aug	52
Y5168	Y5169	3-Jul	-17.39	13.86	Neritic	859	662	115	111	84	75.68	33.8	0.0193	27-Aug	30-Aug	55

Effect of maternal foraging habitat on offspring quality in the loggerhead sea turtle (*Caretta caretta*)
Ecology and Evolution
Hideo Hatase*, Kazuyoshi Omuta, Koutarou Itou and Teruhisa Komatsu
*Corresponding author: hhatase@yahoo.co.jp

tta) nesting at Yakushima Island, Japan, 2015

Number of hatchlings sampled	Hatchling straight carapace length (mm)		Hatchling straight carapace width (mm)		Hatchling body mass (g)	
	Mean	CV	Mean	CV	Mean	CV
15	39.428	0.0202	31.921	0.0255	15.11	0.0364
17	42.578	0.0163	33.488	0.0135	17.15	0.0202
5	41.182	0.0434	33.805	0.0461	15.39	0.0757
19	42.390	0.0157	34.918	0.019	17.85	0.0458
–	–	–	–	–	–	–
11	39.842	0.0239	32.888	0.015	15.25	0.0448
7	42.849	0.0197	34.192	0.0162	17.86	0.0463
2	41.525	0.0171	33.533	0.026	15.47	0.0434
4	40.755	0.0422	32.704	0.0518	16.89	0.0584
10	41.801	0.0265	33.514	0.0264	18.19	0.0504
9	42.518	0.0121	33.180	0.0256	17.44	0.0231
12	40.059	0.0533	33.101	0.0603	16.06	0.0536
16	41.460	0.0149	32.118	0.0212	16.50	0.0366
8	42.954	0.0237	34.863	0.015	18.07	0.031
–	–	–	–	–	–	–
14	40.037	0.025	32.945	0.0338	15.13	0.0328
13	42.518	0.0428	34.115	0.0471	17.96	0.0654
1	41.535	0.0232	33.720	0.0371	16.50	0.0504
20	40.885	0.0254	33.379	0.0189	16.77	0.0668
18	41.262	0.0161	33.943	0.01	16.48	0.0274
