# Supplementary material for: Effect of maternal foraging habitat on offspring quality in the loggerhead sea turtle (Caretta caretta)
Source: Ecol Evol. 2018 Feb 27;8(6):3543–55. doi: 10.1002/ece3.3938 (PMC5869213; doi:10.1002/ece3.3938)
Supplement: Supplementary file 4 [file ECE3-8-3543-s004.pdf]

Table S4. The original data on stable carbon and nitrogen isotope ratios ($\delta^{13}C$ and $\delta^{15}N$) in egg yolks, body size, and egg and hatchling characteristics for 20 loggerhead turtles (*Caretta* [illegible]

Tag number	Tag number	Oviposition date	$\delta^{13}C$ (‰)	$\delta^{15}N$ (‰)	Foraging habitat	Adult straight carapace length (mm)	Adult straight carapace width (mm)	Cltuch size	Number of eggs reburied	Number of emerging hatchlings	Emergence success (%)	Mass (g) for 5 eggs		Date of first emergence	Date of excavation survey	Incubation duration (days)
												Mean	CV			
Y6854	Y6855	8-Jul	-20.29	9.52	Oceanic	737	558	77	76	59	77.63	30.7	0.0315	26-Aug	31-Aug	49
Y7254	Y7255	6-Jul	-19.82	9.84	Oceanic	759	618	106	105	75	71.43	25.66	0.0374	24-Aug	28-Aug	49
Y7331		5-Jul	-20.33	9.97	Oceanic	780	628	119	118	61	51.69	38.1	0.034	22-Aug	26-Aug	48
Y7278	Y7279	6-Jul	-20.05	10.06	Oceanic	790	600	83	82	59	71.95	33.06	0.0444	24-Aug	28-Aug	49
Y7333	Y7334	4-Jul	-19.73	10.09	Oceanic	787	613	89	88	62	70.45	29.96	0.0382	22-Aug	26-Aug	49
Y6767	Y6768	4-Jul	-20.45	10.13	Oceanic	725	623	111	110	80	72.73	26.52	0.034	20-Aug	24-Aug	47
Y6951	Y7293	9-Jul	-20.23	10.28	Oceanic	762	592	95	94	57	60.64	27.92	0.0285	25-Aug	29-Aug	47
Y6388	Y6389	8-Jul	-20.33	10.42	Oceanic	746	601	80	79	42	53.16	29.24	0.0346	26-Aug	31-Aug	49
Y7170	Y7240	5-Jul	-20.00	10.49	Oceanic	759	607	88	87	73	83.91	34.82	0.0284	22-Aug	26-Aug	48
Y7270	Y7280	8-Jul	-19.38	10.73	Oceanic	745	605	92	91	68	74.73	32.18	0.0414	24-Aug	28-Aug	47
Y6866	Y6894	8-Jul	-16.76	11.52	Neritic	847	682	122	121	50	41.32	32.6	0.027	25-Aug	29-Aug	48
Y7343	Y7344	6-Jul	-18.89	12.13	Neritic	842	660	114	111	63	56.76	37.14	0.044	22-Aug	26-Aug	47
Y7364	Y7365	8-Jul	-18.44	12.62	Neritic	833	662	106	105	57	54.29	35.16	0.0189	25-Aug	29-Aug	48
Y6798	Y3412	9-Jul	-17.66	12.76	Neritic	843	670	144	143	63	44.06	31.46	0.0245	24-Aug	28-Aug	46
Y7249	Y7250	4-Jul	-17.70	12.98	Neritic	874	685	135	134	63	47.01	33.84	0.0355	22-Aug	26-Aug	49
Y3276	Y6263	5-Jul	-18.48	13.20	Neritic	839	630	97	96	76	79.17	30.98	0.0656	21-Aug	25-Aug	47
Y6363	Y6364	8-Jul	-17.76	13.74	Neritic	827	670	116	115	56	48.70	33.94	0.0435	27-Aug	31-Aug	50
Y6722	Y6723	5-Jul	-17.90	13.75	Neritic	871	690	119	118	61	51.69	37.42	0.0237	23-Aug	27-Aug	49
Y7188		6-Jul	-17.18	13.84	Neritic	851	688	129	128	80	62.50	33.6	0.0365	23-Aug	27-Aug	48
Y6291	Y6292	4-Jul	-17.52	17.08	Neritic	856	636	102	101	71	70.30	34.62	0.018	22-Aug	26-Aug	49

Effect of maternal foraging habitat on offspring quality in the loggerhead sea turtle (*Caretta caretta*)
Ecology and Evolution

Hideo Hatase*, Kazuyoshi Omuta, Koutarou Itou and Teruhisa Komatsu
*Corresponding author: hhatase@yahoo.co.jp

caretta) nesting at Yakushima Island, Japan, 2016

Number of hatchlings sampled for measuring body size	Hatchling straight carapace length (mm)		Hatchling straight carapace width (mm)		Hatchling body mass (g)		Number of hatchlings sampled for measuring righting response time	Righting response time (s)		Number of hatchlings sampled for measuring righting response propensity	Righting response propensity	
	Mean	CV	Mean	CV	Mean	CV		Mean	CV		Mean	CV
10	39.588	0.0319	32.900	0.0321	13.42	0.0734	10	2.135	0.6464	10	6.00	0.0000
10	38.124	0.0274	30.349	0.0402	12.26	0.0631	8	2.829	0.5235	10	3.20	0.7626
10	41.470	0.0294	34.057	0.0194	16.45	0.0611	10	3.074	0.4997	10	5.40	0.1295
10	42.172	0.0194	33.450	0.0193	17.14	0.0404	10	2.424	0.4832	10	5.80	0.0727
10	40.621	0.0307	32.426	0.0360	14.80	0.0642	10	2.591	0.4159	10	6.00	0.0000
16	39.248	0.0222	31.363	0.0286	13.65	0.0379	6	2.098	0.3971	6	5.67	0.1441
10	39.614	0.0229	32.028	0.0200	13.99	0.0484	10	2.798	0.4841	10	5.20	0.3368
10	39.308	0.0413	32.401	0.0369	14.05	0.0324	10	2.219	0.3999	10	5.80	0.0727
10	42.453	0.0203	33.715	0.0167	17.90	0.0296	10	2.582	0.4710	10	5.30	0.2957
10	40.689	0.0212	33.277	0.0192	16.69	0.0392	10	2.332	0.4861	10	5.90	0.0536
10	41.495	0.0327	33.450	0.0210	16.15	0.0527	10	2.742	0.5874	10	5.10	0.2687
10	43.197	0.0243	34.515	0.0261	19.12	0.0457	10	2.462	0.4755	10	5.50	0.1286
10	42.022	0.0241	34.420	0.0297	18.57	0.0405	10	2.598	0.5447	10	5.30	0.1553
10	40.712	0.0168	33.868	0.0248	16.18	0.0287	10	3.396	0.2301	10	5.00	0.2108
10	42.690	0.0316	33.897	0.0219	17.10	0.0367	10	2.024	0.3054	10	5.60	0.1249
17	41.557	0.0227	32.611	0.0258	15.73	0.0663	7	2.302	0.5768	7	5.29	0.1800
10	41.607	0.0248	33.545	0.0287	16.28	0.0623	9	2.625	0.5311	10	4.30	0.5487
10	42.887	0.0133	34.597	0.0259	18.17	0.0363	10	3.750	0.4725	10	4.60	0.3273
10	42.276	0.0263	33.866	0.0426	17.14	0.0749	8	2.325	0.4294	10	4.20	0.6127
10	40.570	0.0445	33.187	0.0271	16.67	0.0267	10	2.925	0.3867	10	4.80	0.4258
