# Supplementary material for: Effect of maternal foraging habitat on offspring quality in the loggerhead sea turtle (Caretta caretta)
Source: Ecol Evol. 2018 Feb 27;8(6):3543–55. doi: 10.1002/ece3.3938 (PMC5869213; doi:10.1002/ece3.3938)
Supplement: Supplementary file 5 [file ECE3-8-3543-s005.pdf]

Table S5. The original data on growth of 20 hatchlings of the loggerhead turtle (*Caretta caretta*) that were collected at Yakushima Island, Japan, and were reared in the not shown

Maternal tag number	Maternal tag number	$\delta^{13}C$ (‰) in the egg yolk	$\delta^{15}N$ (‰) in the egg yolk	Maternal foraging habitat	Hatchling carapace number	Oviposition date	Date of first emeregence of hatchlings from the nest	Incubation duration (days)	Date of hatchling collection	Hatchling straight carapace length (mm)	Hatchling straight carapace width (mm)	Hatchling body mass (g)	Righting response time (s)
Y7249	Y7250	-17.70	12.98	Neritic	1	4-Jul	22-Aug	49	22-Aug	42.88	33.45	17.3	1.240
Y6767	Y6768	-20.45	10.13	Oceanic	2	4-Jul	20-Aug	47	21-Aug	39.58	32.16	14	2.153
Y6291	Y6292	-17.52	17.08	Neritic	3	4-Jul	22-Aug	49	22-Aug	42	32.46	16.3	2.350
Y7333	Y7334	-19.73	10.09	Oceanic	4	4-Jul	22-Aug	49	22-Aug	41.42	33.38	15.3	2.360
Y7331		-20.33	9.97	Oceanic	5	5-Jul	22-Aug	48	22-Aug	42.02	34.27	16.7	2.187
Y6722	Y6723	-17.90	13.75	Neritic	6	5-Jul	23-Aug	49	23-Aug	43.59	33.49	18.5	5.440
Y3276	Y6263	-18.48	13.20	Neritic	7	5-Jul	21-Aug	47	21-Aug	41.35	33.19	17	
Y7170	Y7240	-20.00	10.49	Oceanic	8	5-Jul	22-Aug	48	22-Aug	43.17	33.65	17.8	1.763
Y7343	Y7344	-18.89	12.13	Neritic	9	6-Jul	22-Aug	47	22-Aug	42.27	33.75	18.4	4.500
Y7278	Y7279	-20.05	10.06	Oceanic	10	6-Jul	24-Aug	49	24-Aug	43.11	32.99	17.2	1.970
Y7188		-17.18	13.84	Neritic	11	6-Jul	23-Aug	48	23-Aug	41.54	32.96	17.5	1.180
Y7254	Y7255	-19.82	9.84	Oceanic	12	6-Jul	24-Aug	49	24-Aug	38.81	31.75	13.3	2.397
Y6854	Y6855	-20.29	9.52	Oceanic	13	8-Jul	26-Aug	49	26-Aug	39.7	33.64	14.9	1.537
Y6363	Y6364	-17.76	13.74	Neritic	14	8-Jul	27-Aug	50	27-Aug	41.43	33.01	16	1.563
Y7270	Y7280	-19.38	10.73	Oceanic	15	8-Jul	24-Aug	47	24-Aug	42.47	34.91	17.9	2.460
Y7364	Y7365	-18.44	12.62	Neritic	16	8-Jul	25-Aug	48	25-Aug	42.2	34.37	19.6	5.023
Y6866	Y6894	-16.76	11.52	Neritic	17	8-Jul	25-Aug	48	25-Aug	41.39	33	15.7	2.800
Y6388	Y6389	-20.33	10.42	Oceanic	18	8-Jul	26-Aug	49	26-Aug	39.54	32.76	14	2.580
Y6798	Y3412	-17.66	12.76	Neritic	19	9-Jul	24-Aug	46	24-Aug	40.49	34.2	16	4.730
Y6951	Y7293	-20.23	10.28	Oceanic	20	9-Jul	25-Aug	47	25-Aug	39.08	32.44	14.5	2.277

Effect of maternal foraging habitat on offspring quality in the loggerhead sea turtle (*Caretta caretta*)
Ecology and Evolution
Hideo Hatase*, Kazuyoshi Omuta, Koutarou Itou and Teruhisa Komatsu
*Corresponding author: hhatase@yahoo.co.jp

Minamichita Beachland Aquarium, Aichi Prefecture, Japan, 2016. Growth data of dead turtles are

Righting response propensity	Date of 2nd measurement	Straight carapace length (mm)	Straight carapace width (mm)	Body mass (g)	Date of 3rd measurement	Straight carapace length (mm)	Straight carapace width (mm)	Body mass (g)
6	31-Oct	58.9	50.54	39				
6								
6	31-Oct	60.37	53.15	39				
6	31-Oct	61.24	54.9	47	19-Dec	108.6	97.83	294
6								
1	31-Oct	64.33	55.86	56	19-Dec	104.03	91.64	238
	31-Oct	58.21	51.38	36				
6	31-Oct	58.64	51.84	34				
6								
6	31-Oct	52.44	45.4	28				
6	31-Oct	55.52	50.85	31				
3								
6								
6	31-Oct	55.57	48.92	36				
6	31-Oct	61.26	57.35	53				
6	31-Oct	60.5	55.72	54	19-Dec	95.96	87.48	210
6	31-Oct	49.6	44.56	26				
6								
5	31-Oct	62.42	60.33	52	19-Dec	92.06	79.2	165
6								
